# Semiflexible Polymers in the Bulk and Confined by Planar Walls

**DOI:** 10.3390/polym8080296

**Published:** 2016-08-10

**Authors:** Sergei A. Egorov, Andrey Milchev, Kurt Binder

**Affiliations:** 1 Department of Chemistry, University of Virginia, Charlottesville, VA 22901, USA; 2 Institut für Physik, Johannes Gutenberg Universität Mainz, 55099 Mainz, Germany; kurt.binder@uni-mainz.de; 3 Institute for Physical Chemistry, Bulgarian Academia of Sciences, 1113 Sofia, Bulgaria; milchev@ipc.bas.bg

**Keywords:** semiflexible polymers, isotropic-nematic behavior, molecular dynamics, density functional theory

## Abstract

Semiflexible polymers in solution under good solvent conditions can undergo an isotropic-nematic transition. This transition is reminiscent of the well-known entropically-driven transition of hard rods described by Onsager’s theory, but the flexibility of the macromolecules causes specific differences in behavior, such as anomalous long wavelength fluctuations in the ordered phase, which can be understood by the concept of the deflection length. A brief review of the recent progress in the understanding of these problems is given, summarizing results obtained by large-scale molecular dynamics simulations and density functional theory. These results include also the interaction of semiflexible polymers with hard walls and the wall-induced nematic order, which can give rise to capillary nematization in thin film geometry. Various earlier theoretical approaches to these problems are briefly mentioned, and an outlook on the status of experiments is given. It is argued that in many cases of interest, it is not possible to describe the scaled densities at the isotropic-nematic transition as functions of the ratio of the contour length and the persistence length alone, but the dependence on the ratio of chain diameter and persistence length also needs to be considered.

## 1. Introduction

There exist many macromolecules where local stiffness is relatively large, i.e., they have a persistence length ($\ell_{p}$) much larger than the diameter (*d*) of the effective monomeric units. Such semiflexible polymers may exhibit liquid-crystalline order [1,2], are of interest as building blocks of various complex soft materials [3,4] and also occur as ingredients of biological matter [5,6,7,8,9,10]. Here, we shall disregard all of these very interesting applications, focusing only on the generic case where these macromolecules exist in semidilute or concentrated solutions under good solvent conditions. However, applying a coarse-grained description, solvent molecules are not explicitly considered; monomeric units then exhibit an effective repulsive interaction, and the monomer concentration *ρ*, as well as the contour length *L* of the chains, are the basic parameters to control the properties of such systems. A characteristic feature, observed for large enough values of the ratio $\ell_{p}/d$, is the onset of nematic long range order [11,12,13,14,15,16,17,18,19,20,21,22]. The simplest case of such lyotropic liquid crystalline systems is solutions of hard rods, such as tobacco mosaic viruses in water [23], a problem that inspired Onsager [24] to develop his famous theory of purely entropically-driven phase transitions [25,26], resulting from the competition of translational and the orientational entropy contributions of the rods. The present problem reduces to this hard rod limit if L/d≫1 and $\ell_{p}$ is taken to infinity. It then suffices to consider the free energy of the system on the level of an approximation where only the second virial coefficient is kept [24].

Describing semiflexible polymers in the limit of the Kratky–Porod worm-like chain model [27], this Onsager-type treatment has been carried over to the description of the isotropic to nematic transition by Khokhlov and Semenov [12,13,14], Odijk [15,16], Chen [20] and others. In the isotropic phase, it is implied that the mean-squared end-to-end distance $\langle R_{e}^{2}\rangle$ of the chains is described by the Kratky–Porod model [27],

(1) $$\langle R_{e}^{2}\rangle =2\ell_{p}L\left\{1-\frac{\ell_{p}}{L}\left[1-\exp\left(-L/\ell_{p}\right)\right]\right\},$$

which reduces to $\langle R_{e}^{2}\rangle =L^{2}$ in the rod-limit ($L\ll \ell_{p}$), but implies Gaussian chain behavior ($\langle R_{e}^{2}\rangle =\;2\ell_{p}L=l_{K}L$, $l_{K}$ being the Kuhn length [28]) in the opposite limit. Obviously, the swelling of the coils due to the excluded volume interaction between the monomers [29] is neglected, although excluded volume matters in the nematic phase. The monomer concentration $\rho_{i}$, where the nematic order starts, is predicted to scale as follows [12,13,14,15,16,20]:

(2) $$\begin{array}{c} \rho_{i}\ell_{p}/d\propto \left\{\begin{array}{cc} const, & L\gg \ell_{p}\\ \ell_{p}/L, & L\ll \ell_{p} \end{array}\right.. \end{array}$$

Note that the isotropic to nematic transition is weakly of first order, but the width of the two-phase coexistence region ($\rho_{i}<\rho <\rho_{n}$, only for $\rho \geq \rho_{n}$, the system exhibits uniform nematic long-range order) is predicted to be rather narrow [12,13,14,15,16,20].

In many cases of practical interest, however, the system is not in the limit $d/\ell_{p}\to 0$, then, $\rho_{i}$ (and $\rho_{n}$) are not extremely small, and the assumption that only the second virial coefficient matters is not justified. Various attempts have been made to estimate the free energy going beyond the second virial coefficient [17,18,19,21], but unfortunately, many of these treatments require somewhat ad hoc assumptions and/or uncontrolled approximations. As a result, various approaches have led to different results not in agreement with each other, and hence, no general consensus on how to go beyond Equation (2) has emerged.

The present authors have taken a new approach towards these issues, combining large-scale molecular dynamics (MD) simulations [30,31] (feasible via the use of graphics processing units (GPUs) [32,33]) with classical density functional theory (DFT). The latter approach is considered in general the most powerful version of mean field theory to describe ordering phenomena in condensed matter and has seen broad and significant progress in recent years [34,35,36,37], but for the most part, this technique is concerned with simple liquids (described by point-like particles interacting via isotropic potentials). Both the generalization to anisotropic particles, whose interactions depend on their mutual orientations (see, e.g., [38]) and the generalization to flexible (e.g., [39]) and semiflexible polymers (e.g., [40,41]) are highly nontrivial and are still subjects of ongoing research. While for simple fluids, the basic object of the theory is the spatially nonuniform density ρ(r), for semiflexible macromolecules, one needs to operate with a function $\rho_{\mathrm{mol}}\left(r,\omega \right)$, which depends not only on the particle position r, but also on the local orientation *ω* (*ω* is a shorthand notation for the polar angles *θ*, ϕ of the molecular bonds). Thus, our recent work [42,43,44,45] not only goes far beyond previous simulation approaches (e.g., [46,47,48,49,50]) by simulating much larger systems (up to 700,000 monomers) and varying parameters, such as *L* and $\ell_{p}$ over much wider ranges than were accessible in the earlier work, but we also extend the DFT methodology for semiflexible polymers by considering simultaneously both of their orientational distribution and spatial inhomogeneity. The comparison with MD, in turn, provides a stringent test of the conditions under which these extensions are accurate and elucidates the reasons for its limitations. A third new ingredient of the work [42,43,44,45] is the use of the concepts of the cylindrical confinement of semiflexible polymers [51,52,53,54] to interpret the anomalous fluctuations of the semiflexible chains, on the length scale of the deflection length *λ* along the contour. These long wavelength collective deflections of bundles of neighboring chains are soft modes that are not implicit in the DFT framework. At this point, we also note that these fluctuations are also not correctly included when one models the semiflexible chains by lattice models (which otherwise may be advantageous from the computational point of view [55,56,57,58,59,60,61,62]). The present off-lattice models thus have also a particular advantage when one wishes to test theories of the interaction of semiflexible polymers with confining walls (e.g., [63,64,65,66]).

The outline of the remainder of this review is as follows. In Section 2, we summarize the models on which the MD and DFT work is based, outline the basic aspects of the computational approaches that are used and remind the reader about the basic aspects of earlier work. In Section 3, we summarize the main features of the behavior of semiflexible polymers in the bulk, considering the variation of persistence length, contour length and monomer density, and also present some comparisons with earlier theories and experiments. Section 4 gives some results on the interaction of semiflexible chains with repulsive walls, including a discussion of capillary nematization in thin films. Section 5 gives a brief summary and an outlook.

## 2. Coarse-Grained Models for Semiflexible Polymers

### 2.1. Molecular Dynamics

There is much recent effort (e.g., [67,68,69,70]) in trying to construct simulation models of macromolecules that address specific effects of their chemical structure. This is not our focus here; we are rather concerned with generic models that address only the general features of lyotropic solutions of semiflexible macromolecules. Thus, the MD work is based on the standard bead-spring model [71,72] amended by a bond-angle potential (see, e.g., [73]), where consecutive effective monomers along the backbone of the chain (the beads) interact with the finitely-extensible nonlinear elastic (FENE) potential [71,72] $U^{\mathrm{FENE}}\left(r\right)$ plus the repulsive part of the Lennard–Jones potential (Weeks–Chandler–Andersen [74] potential $U^{\mathrm{WCA}}\left(r\right)$). Explicitly, these potentials are defined as follows:

(3) $$U^{\mathrm{FENE}}\left(r\right)=-0.5kr_{0}^{2}\ln\left[1-{\left(\frac{r}{r_{0}}\right)}^{2}\right],\;r<r_{0},$$

while $U^{\mathrm{FENE}}\left(r>r_{0}\right)\equiv 0$, and:

(4) $$U^{\mathrm{WCA}}\left(r\right)=4\varepsilon \left[{\left(\frac{\sigma }{r}\right)}^{12}-{\left(\frac{\sigma }{r}\right)}^{6}+\frac{1}{4}\right],\;r<r_{c}=2^{1/6}\sigma \;,$$

and $U^{\mathrm{WCA}}\left(r>r_{c}\right)\equiv 0$. The parameters of the FENE potential are taken as $r_{0}=1.5\sigma$ and $k=\;30\varepsilon /\sigma^{2}$ as usual, and then, the total binding potential $U^{\mathrm{FENE}}\left(r\right)+U^{\mathrm{WCA}}\left(r\right)$ yields a rather sharp minimum at the effective bond length $\ell_{b}\approx 0.97\sigma$. The contour length of a chain containing *N* beads is $L\approx \;\left(N-\;1\right)\ell_{b}$.

We stress that the potential $U^{\mathrm{WCA}}\left(r\right)$ is also applied between any two effective monomers in the system, not only bonded ones. Note that the solvent molecules are not explicitly considered; hence, the model corresponds to very good solvent conditions, and the effective monomer diameter *d* can be taken as d=σ. Finally, the bond-bending potential is:

(5) $$U^{\mathrm{bend}}\left(\theta_{ijk}\right)=\epsilon_{b}\left[1-\cos\left(\theta_{ijk}\right)\right]\approx \epsilon_{b}\theta_{ijk}^{2}/2,$$

where the energy parameter $\epsilon_{b}$ controls the chain stiffness and the angle $\theta_{ijk}$ is the bond angle between the two subsequent bond vectors, $a_{i}=r_{j}-r_{i}$ and $a_{j}=r_{k}-r_{j}$.

Choosing units such that σ=1 sets the scale of length and ε=1 sets the scale of energy (the thermal energy being chosen $k_{B}T=1$ throughout), the parameters chain length *N* and bending energy $\epsilon_{b}$, together with the monomer density *ρ*, are the control parameters of the model. We also note that $\epsilon_{b}$ is simply related [75] to the persistence length $\ell_{p}$ via the average of $\cos\theta_{ijk}$:

(6) $$\ell_{p}/\ell_{b}=-1/\ln\langle \cos\theta_{ijk}\rangle .$$

The definition in Equation (6) implies (via Equation (5)) that the persistence length plays the role of a coupling constant in the effective Hamiltonian of worm-like chains (continuous version of the Kratky–Porod model). Other definitions of the persistence length exist, e.g., [76], but have some clear disadvantages, e.g., they are not applicable in d=2 dimensions [75,77].

Note that the approximate equalities in Equations (5) and (6) hold for $\epsilon_{b}\geq 2$, and then, $U^{\mathrm{bend}}\left(\theta_{ijk}\right)$ is harmonic in $\theta_{ijk}$; hence $\langle U^{\mathrm{bend}}\left(\theta_{ijk}\right)\rangle =k_{B}T/2=1/2$, and hence, $\langle \theta_{ijk}^{2}\rangle =1/\epsilon_{b}$; and thus, $\ell_{p}/\ell_{b}=\;\epsilon_{b}$ (in our units). We stress that for our problem, the notion of persistence length makes sense only as a description of the local chain stiffness [75], and it must not be related to the asymptotic decay of bond-angle correlations via the textbook formula [28]:

(7) $$\langle \cos\theta_{i,i+s}\rangle \equiv \langle a_{i}\cdot a_{i+s}\rangle /\langle a_{i}^{2}\rangle =\exp\left(-s\ell_{b}/\ell_{p}\right).$$

While Equation (7) reduces to Equation (6) for s=1, it would fail for large *s* in general, apart from non-interacting phantom chains [28,75]. Due to excluded volume effects, the asymptotic decay of $\langle \cos\theta_{i,i+s}\rangle$ is of power law form, as discussed extensively elsewhere [75], for $\ell_{p}/\ell_{b}<s\ll N$, in the isotropic phase of the solution. In the nematic phase, we expect that $\langle \cos\theta_{i,i+s}\rangle$ develops a plateau independent of *s*, related to the nematic order parameter.

MD simulations are done at constant density, choosing N chains of length *N* in a box of volume $V_{\mathrm{box}}$, and hence:

(8) $$\rho =NN/V_{\mathrm{box}}.$$

In order to study the phase behavior in the bulk, one chooses a box of simple cubic shape (linear dimension $L_{\mathrm{box}}=V_{\mathrm{box}}^{1/3}$) and periodic boundary conditions in the *x*, *y* and *z* directions. When we are interested in the effect of repulsive walls, one chooses $V_{\mathrm{box}}=L_{x}^{2}L_{z}$, with two repulsive walls at z=0 and $z=L_{z}$, and periodic boundary conditions only in *x* and *y* directions. The repulsive potential due to the walls then simply is:

(9) $$U_{\mathrm{wall}}=U^{\mathrm{WCA}}\left(z\right)+U^{\mathrm{WCA}}\left(L_{z}-z\right).$$

When one chooses $L_{z}$ very large, in particular $L_{z}\gg L$, for densities corresponding to the density of the bulk isotropic phase $\rho_{b}$, the two walls can be treated as non-interacting, and thus, one can relate the behavior observed in such simulations to wall effects on semi-infinite solutions. In the case where the distance $L_{z}$ between the walls and the contour length *L* are comparable, this is not the case, and the problem of “capillary nematization” (well-known for hard-rod fluids; see, e.g., [78]) must be addressed.

In the MD simulations, configurations of the chains in the simulated volume are generated by solving numerically Newton’s equation of motion for the beads, using the velocity Verlet algorithm [30,31], and applying a “Langevin thermostat” to fix $k_{B}T=1$:

(10) $$m\frac{d^{2}r_{n}}{dt^{2}}=F_{\mathrm{tot}}\left(r_{n}\right)-\gamma \frac{dr_{n}}{dt}+F_{n}^{\mathrm{rand}}\left(t\right),$$

where m(=1) is the mass of an effective monomer having position $r_{n}$, *t* is the time along the generated system trajectory through phase space, $F_{\mathrm{tot}}\left(r_{n}\right)$ describes the total force obtained as the gradient of the total potential, due to $U^{\mathrm{FENE}}$, $U^{\mathrm{WCA}}$, $U^{\mathrm{bend}}$ (and $U_{\mathrm{wall}}$ in the presence of repulsive walls) acting on the considered bead. The friction coefficient γ(=0.25) is related to the random force $F_{n}^{\mathrm{rand}}\left(t\right)$ by the fluctuation-dissipation theorem:

(11) $$\langle F_{n}^{\mathrm{rand}}\left(t\right)F_{n^{\prime }}^{\mathrm{rand}}\left(t^{\prime }\right)\rangle =6k_{B}T\gamma \delta_{nn^{\prime }}\delta \left(t-t^{\prime }\right).$$

For the present choice of parameters, the MD time unit is $\tau_{\mathrm{MD}}=\sqrt{m\sigma^{2}/\epsilon }=1$, and to actually solve Equation (10), discrete time increments δt=0.01 are used, employing the HooMD-blue software package [32,33] on various GPUs. From the obtained trajectories, various quantities of interest, such as pressure, chain linear dimensions and orientational order parameters, can be deduced.

### 2.2. Density Functional Theory

Starting with the seminal work of Onsager [24], DFT has been the basic theoretical approach to deal with entropically-driven phase transitions. We choose here the nomenclature to reserve the notation DFT for the implementations developed by us recently [42,43,44,45] and refer to the earlier DFT theories by the names of the respective authors. For implementing DFT, it is more convenient to describe the polymer molecules as necklaces of tangent hard spheres of diameter *σ*, but one can choose the same bending potential as done in MD, Equation (5). Thus, it is also implied that non-bonded monomers interact with the simple hard-sphere potential; and when one includes in the model the interaction of monomers with repulsive walls, the latter are also taken to be hard walls: $U_{\mathrm{wall}}\left(z\leq 0\right)=\infty$, $U_{\mathrm{wall}}\left(z\geq L_{z}\right)=\infty$ and $U_{\mathrm{wall}}\left(0<z<L_{z}\right)=0$.

However, we first discuss the DFT implementation used for studying the phase behavior in the bulk [42,43]. The density $\rho_{\mathrm{mol}}\left(r,\omega \right)$ is then taken as the product of the chain density $\rho_{\mathrm{mol}}=N/V$ and the orientational distribution function f(ω) [41,79,80], where f(ω)=1/4π in the isotropic phase. The Helmholtz free energy then is decomposed, as usual, into ideal and excess terms:

(12) $$F\left(\rho_{\mathrm{mol}}\left(r,\omega \right)\right)=F_{id}\left(\rho_{\mathrm{mol}}\left(r,\omega \right)\right)+F_{exc}\left(\rho_{\mathrm{mol}}\left(r,\omega \right)\right).$$

For a bulk system, the ideal term is, after the integration over *ω*:

(13) $$F_{\mathrm{id}}\left(\rho_{\mathrm{mol}}\right)/Nk_{B}T=\ln\left(\rho_{\mathrm{mol}}\right)-1+\int d\omega f\left(\omega \right)\ln\left[4\pi f\left(\omega \right)\right].$$

Obviously, the last term (the ideal orientational entropy) is zero in the isotropic phase, but nontrivial in the nematic phase.

When infinitely long rigid rods are considered, Onsager’s theory [24] readily yields the second term in Equation (12), based on the second virial coefficient term in the virial expansion:

(14) $$F_{\mathrm{exc}}^{\mathrm{Ons}}\left(\rho_{\mathrm{mol}}\right)/Nk_{B}T=\frac{1}{2}\rho_{\mathrm{mol}}\int d\omega \int d\omega^{\prime }f\left(\omega \right)f\left(\omega^{\prime }\right)V_{\mathrm{exc}}\left(\omega ,\omega^{\prime }\right)=\frac{\rho_{\mathrm{mol}}}{2}\langle V_{\mathrm{exc}}\rangle .$$

Here, $V_{\mathrm{exc}}\left(\omega ,\omega^{\prime }\right)$ is the excluded volume for two rods with orientations *ω* and $\omega^{\prime }$. However, here, we wish to study semiflexible polymers of finite length rather than rigid rods of infinite length. To go beyond the second virial approximation, a common way is to introduce a rescaling prefactor [81,82] $a_{\mathrm{resc}}$:

(15) $$F_{\mathrm{exc}}\left(\rho_{\mathrm{mol}}\right)/Nk_{B}T=\frac{1}{2}a_{\mathrm{resc}}\langle V_{\mathrm{exc}}\rangle .$$

Egorov et al. [42,43] applied two different choices for $a_{\mathrm{resc}}$. The first one, leading to a version of DFT called DFT-CS (DFT-Carnahan-Starling), is based [81,82] on the Carnahan–Starling [83] equation of state for the simple hard-sphere fluid:

(16) $$a_{\mathrm{resc}}=\rho_{\mathrm{mol}}\frac{4-3\eta }{4{\left(1-\eta \right)}^{2}},\;\eta =\rho_{b}\pi \sigma^{3}/6.$$

Note that *η* is just the monomer packing fraction, and $\rho_{b}=N\rho_{\mathrm{mol}}$. Equation (16) completely disregards chain connectivity, but has the merit that it reduces to the Onsager limit for η→0.

The second choice [84] is based on an (approximate) form for the excess free energy $F_{\mathrm{exc}}^{\mathrm{iso}}\left(\rho_{\mathrm{mol}}\right)$ of the polymeric fluid in the isotropic state (but does not reproduce the Onsager limit for η→0). While various expressions (see, e.g., [40,85,86]) are known for $F_{\mathrm{exc}}^{\mathrm{iso}}\left(\rho_{\mathrm{mol}}\right)$ for flexible polymers, $F_{\mathrm{exc}}^{\mathrm{iso}}\left(\rho_{\mathrm{mol}}\right)$ is not known for semiflexible polymers. Therefore, the generalized Flory dimer (GFD) equation of state [87] was used [42,43] to compute the rescaling factor $a_{\mathrm{resc}}$ in this formulation [84], to obtain a version of DFT that was termed DFT-GFD.

However, both versions require the knowledge of $V_{\mathrm{exc}}\left(\omega ,\omega^{\prime }\right)$ for semiflexible polymers, which is not known analytically, of course. Therefore, Egorov et al. [42,43] used an empirical expression proposed by Fynewever and Yethiraj [41] obtained by fitting the data from Monte Carlo simulations of a system containing just two semiflexible chains. Of course, from this description, it is evident that in spite of the rigorous foundation [37] of DFT in terms of a variational principle for the grand potential as a functional of the average particle density, the practical implementation of DFT in the present application is hampered by various more or less empirical assumptions and approximations whose accuracy it is difficult to assess a priori. The main motivation for the two versions of DFT that are presented here is that they are validated (at least qualitatively) over a fairly broad range of parameters by comparison with the MD results. We emphasize that in this treatment, we have effectively accounted for the effects of higher-order terms in the virial expansion, at least approximately.

Of course, when F(ρ) has been computed for the isotropic and nematic phases, the chemical potential and pressure for both phases can be easily obtained, and the phase diagram for the isotropic-nematic transition can be constructed. The order parameter in the nematic phase follows from the molecular orientational distribution function, recalling that *ω* is a shorthand notation for (θ,ϕ):

(17) $$S=\int d\omega f\left(\omega \right)\left(\frac{3}{2}\cos^{2}\theta -\frac{1}{2}\right).$$

When one now considers the extension of the theory to confinement by planar walls [44,45], the starting point is still Equation (12), but $\rho_{\mathrm{mol}}\left(r,\omega \right)$ can no longer be taken as $\rho_{\mathrm{mol}}\left(r,\omega \right)=\rho_{\mathrm{mol}}f\left(\omega \right)$; rather, we must have:

(18) $$\rho_{\mathrm{mol}}\left(r,\omega \right)=\rho_{\mathrm{mol}}\left(z,\omega \right)=\rho_{\mathrm{iso}}\left(z\right)f\left(z,\omega \right),$$

restricting attention to the case where the solution far from the walls is in the isotropic phase. We note here that while in the bulk DFT calculations (which do not resolve individual monomers on the chain), *ω* describes the orientation of the entire chain [42,43], the DFT calculations under planar confinement are performed on a monomer-resolved level, and f(z,ω) for the molecule is obtained by averaging the corresponding orientational distribution functions for the individual bonds over all of the bonds in the chain [44,45].

The task is now the minimization of the grand potential Ω:

(19) $$\Omega \left(\rho_{\mathrm{mol}}\left(z,\omega \right)\right)=F\left(\rho_{\mathrm{mol}}\left(z,\omega \right)\right)+\int_{0}^{L_{z}}dz\rho_{\mathrm{iso}}\left(z\right)\int d\omega f\left(z,\omega \right)\left[V_{\mathrm{ext}}^{\mathrm{mol}}\left(z,\omega \right)-\mu \right],$$

where *μ* is the polymer chemical potential and $V_{\mathrm{ext}}^{\mathrm{mol}}\left(z,\omega \right)$ is the external potential due to the two hard walls acting on the polymer molecules. The ideal term in Equation (12) for this case is still known exactly [44], while the excess term is split into an isotropic part $F_{\mathrm{exc}}^{\mathrm{iso}}\left(\rho_{\mathrm{iso}}\left(z\right)\right)$ and an orientational part. As a generalization of Equation (14), one needs a model for the excluded volume term $V_{\mathrm{exc}}\left(r,r^{\prime },\omega ,\omega^{\prime }\right)$, which is both spatially and orientationally dependent. However, this term is known explicitly only for two rigid rods under planar confinement [88]; for two semiflexible polymers, we make a heuristic approximation

(20) $$V_{\mathrm{exc}}\left(r,r^{\prime },\omega ,\omega^{\prime }\right)\approx \delta \left(r-r^{\prime }\right)V_{\mathrm{exc}}\left(\omega ,\omega^{\prime }\right),$$

using again the same approximation for $V_{\mathrm{exc}}\left(\omega ,\omega^{\prime }\right)$ as used for the bulk [41]. We note here that a better approximation would have been obtained by averaging the corresponding Mayer function over *x* and *y* variables. However, we do not pursue this approach here, because even within the crude approximation of Equation (20), the minimization of Equation (19) with respect to the two functions $\rho_{\mathrm{iso}}\left(z\right)$ and f(z,ω) is delicate and requires substantial numerical effort (see [44], for details). The generalization of Equation (17) then is:

(21) $$S\left(z\right)=2\pi \int_{0}^{\pi }d\theta f\left(z,\theta \right)\left(\frac{3}{2}\cos^{2}\theta -\frac{1}{2}\right),$$

to obtain the order parameter as a function of distance from the walls. Here, *θ* is understood as a polar angle with the *z*-axis perpendicular to the walls (unlike Equation (17), where nematic order is assumed and *θ* is measured relative to the director), since we focus here on the orientational order induced by the walls. Thus, S(z)=0 corresponds to random chain orientation (recall that the orientational distribution function f(z,ω) is defined as an average over all of the bonds in the molecule), while S(z)=-0.5 corresponds to perfect alignment of the chain parallel to the wall. Note that a particular bonus of DFT is that it yields the free energy explicitly, which will depend on $L_{z}$ due to the wall excess contributions. The dimensionless surface tension hence can be obtained when the corresponding bulk term $L_{z}f_{\mathrm{bulk}}$ is subtracted ($f_{\mathrm{bulk}}$ is the bulk free energy density, and it is assumed that *F* is normalized per unit area of the walls):

(22) $$\frac{\gamma_{\mathrm{wall}}\sigma^{2}}{k_{B}T}=\frac{\sigma^{2}}{2k_{B}T}\left(F-L_{z}f_{\mathrm{bulk}}\right)=\frac{\sigma^{2}}{2k_{B}T}\left(\Omega -L_{z}\Omega_{\mathrm{bulk}}\right),$$

where Ω and $\Omega_{\mathrm{bulk}}$ are the grand potential density and its bulk value, respectively.

### 2.3. A Brief Review of Earlier Theories

Onsager’s theory for the isotropic-nematic transition of rigid rods [24] was extended to semiflexible polymers by Khokhlov and Semenov [12,13,14] and Odijk [15,16]. Some approximations made by these authors are avoided in the treatment of Chen [20], and hence, we only sketch this latter treatment here briefly. The free energy per chain of the system is written as a sum of three terms ($c=NL^{2}d/V_{\mathrm{box}}$ is a dimensionless number density):

(23) $$\frac{F}{Nk_{B}T}=\ln\left(\frac{4\pi c}{Q}\right)-\int d\omega f\left(\omega \right)U_{\mathrm{MF}}\left(\omega \right)+c\int d\omega \int d\omega^{\prime }\left|\sin\alpha \right|f\left(\omega \right)f\left(\omega^{\prime }\right).$$

Here, *Q* is the partition function of a semiflexible chain, and hence, the first two terms on the right-hand side represent the entropy of a semiflexible polymer. The last term represents the excluded volume interaction between the two chains; *α* is the angle between two unit vectors pointing at *ω* and $\omega^{\prime }$. The mean field $U_{\mathrm{MF}}\left(\omega \right)$ represents the averaged orienting effect of the neighboring chains on the considered chain and needs to be determined self-consistently. Thus, from Equation (23), it is clear that excluded volume is only dealt with on the level of the second virial coefficient, and fluctuations beyond the mean-field approximation are neglected.

The explicit computation of *F* is based on the Kratky–Porod [27] model for semiflexible chains, using a functional integral approach [89], where the semiflexible polymer is described by a continuous space curve, specified by its tangent unit vector n(t), 0≤t≤1 (t=0, 1 correspond to the free ends of the polymer chain). The statistical probability P{n(t)} of such a chain configuration is [89]:

(24) $$P\left\{n\left(t\right)\right\}=\exp\left\{-\frac{1}{4}\frac{\ell_{p}}{L}\int_{0}^{1}{\left(\frac{dn(t)}{dt}\right)}^{2}dt\right\}.$$

There is no excluded volume considered in Equation (24), and thus, Equation (24) can be shown to yield Equation (1). One introduces a partition function q(t,ω) of a chain of length tL that has the final end-point pointing at orientation *ω*, which satisfies the equation [90]:

(25) $$\frac{\partial q}{\partial t}=\left\{\frac{L}{\ell_{p}}\nabla_{n}^{2}-U_{\mathrm{MF}}\left(\omega \right)\right\}q,$$

with q(t=0,ω)=1, and is related to f(ω) via:

(26) $$f\left(\omega \right)=\frac{1}{Q}\int_{0}^{1}dsq\left(s,\omega \right)q\left(1-s,\omega \right),$$

where Q=∫dωq(1,ω). Of course, this set of coupled self-consistent nonlinear equations cannot be solved analytically, but requires a numerical iteration procedure [20]. Khokhlov and Semenov [12,13,14] and Odijk [15,16] have avoided this problem by using an approximate variational method to minimize *F*, employing trial functions f(ω) with a single variational parameter. We shall briefly discuss the differences between these results when we compare them to the results of DFT and MD methods [42,43]. Here, we also mention the extension of the Khokhlov–Semenov theory due to Hentschke [17], who attempted to improve the treatment of the orientational free energy of semiflexible polymers by accounting more carefully for the excluded volume effects, while DuPré and Yang [19] attempted to go beyond the second virial coefficient in their treatment of the excluded volume interaction, in order to enable the study of liquid-like densities. A related goal was addressed by Sato and Teramoto [18,21], who extended the scaled particle theory [91,92] to renormalize the strength of the excluded volume interaction in Equation (23), modifying also the ideal gas-like term. With respect to f(ω), Sato and Teramoto [18,21] continued to use trial functions similar as done by Khokhlov and Semenov [12,13,14]. Unfortunately, the accuracy of these various extensions of the Khokhlov–Semenov theory is hard to assess a priori, and hence, this problem is outside of the scope of the present brief review.

## 3. Phase Behavior and Nematic Order of Semiflexible Macromolecules in Bulk Solution

### 3.1. The Isotropic-Nematic Transition and Its Dependence on $\ell_{p}$, L, and d

**Figure 1:**
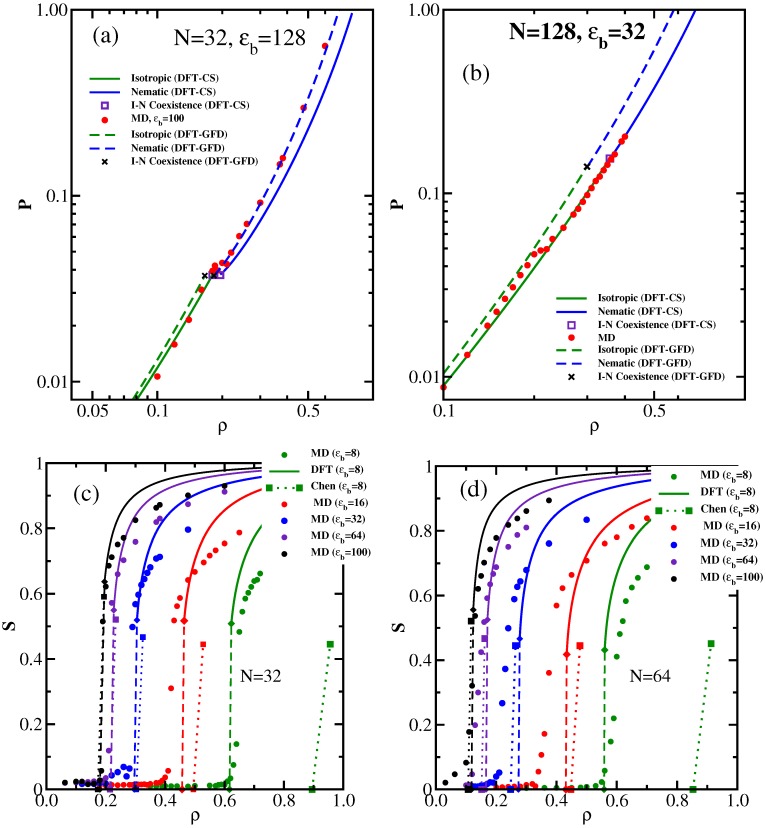
(**a**) Pressure *P* vs. density *ρ* for semiflexible chains with N=32 beads and $\epsilon_{b}=128$. Due to the choice of units σ=1, ϵ=1, $k_{B}T=1$, both *P* and *ρ* are dimensionless. Circles represent MD data, while curves denote the corresponding DFT-CS and DFT-GFD predictions, as indicated. Open squares and crosses indicate coexistence conditions; (**b**) Same as (a), but for N=128 and $\epsilon_{b}=32$; (**c**) Order parameter as a function of density for semiflexible chains of length N=32 and various choices of $\epsilon_{b}$, as indicated. Circles are MD results, curves are DFT-CS predictions for S(ρ), ending at $S\left(\rho_{n}\right)=S_{c}$ (diamonds), while the linear variation in the I-N coexistence region is shown by dashed straight lines. The corresponding predictions from Chen [20] for the I-N coexistence region are shown by dotted straight lines, ending in squares; (**d**) Same as (c), but for N=64. Reproduced from [43] with permission from the Royal Society of Chemistry.

We discuss here the variation of system properties as a function of the density *ρ* of the effective monomers, Equation (8). Since the isotropic-nematic transition is of the first order, one expects to find a two-phase coexistence region from $\rho =\rho_{i}$ to $\rho =\rho_{n}$, at a coexistence pressure $p=p_{\mathrm{coex}}$. The order parameter *S* (Equation (17)) should vary linearly from $S(\rho_{i})=0$ to $S\left(\rho_{n}\right)=S_{c}$, due to the lever rule of two-phase coexistence. While this behavior is consistent with the DFT calculations, where one treats the isotropic and nematic phases separately and locates the transition from the condition that both the chemical potentials of coexisting phases and their pressures must be equal, MD work in the canonical constant density ensemble is hampered by finite size effects, and so, in the pressure vs. density isotherms (Figure 1a,b), the transition only shows up as a small wiggle, rather than as a strictly horizontal line. Furthermore, in the variation of the order parameter *S* with density finite size, effects cause a “finite size tail” [93] in the disordered phase, a well-known effect from simulation studies of other phase transitions. Therefore, the kink singularities in the S(ρ) vs. *ρ* curve at $\rho =\rho_{i}$ and $\rho =\rho_{n}$ are not seen due to the finite size rounding. An interesting observation is the fact that the order parameter predicted by DFT reaches saturation much faster than found in MD (Figure 1c,d). In this context, it is interesting to note that for the nematic order parameter of rod-like molecules described by the hard Gaussian overlap fluid, also an overestimation of the order parameter by DFT in comparison with simulation results has been observed [94]. We shall discuss the behavior of the order parameter and its relation to the deflection length in Section 3.2 below.

Testing now the quantitative accuracy of the two versions of DFT introduced in Section 2.2 for the equation of state (Figure 1a,b) by comparing the *p* vs. *ρ* isotherms to the corresponding MD data, a clear trend as to which version works better does not emerge. For large values of $\epsilon_{b}$, the width of the I-N (Isotropic-Nematic) coexistence region is so small, that on the scale of Figure 1b, it is hardly resolved. Of course, a perfect quantitative agreement between MD and DFT should not even be expected, since the underlying chain models differ slightly. From Figure 1c,d, we see that the theory of Chen [20] predicts the I-N coexistence region rather well as long as the transition densities are small enough (ρ<0.3), but becomes more and more inaccurate the larger the transition densities get. Such a failure is expected, of course, since the theories [12,13,14,15,16,20] describe the chain interactions only on the level of the second virial coefficient, as discussed in Section 2.2.

**Figure 2:**
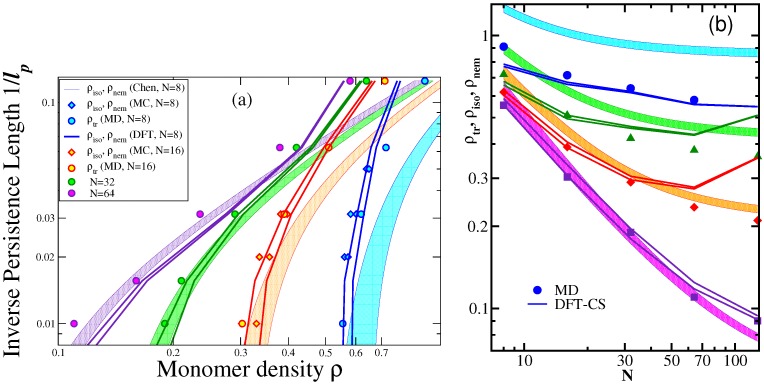
(**a**) Predictions for the I-N transition densities from [20] compared to MD and DFT results [43], in a log-log plot of inverse persistence length vs. density. Different symbols and different colors indicate the different chain lengths *N* = 8, 16, 32 and 64, respectively, as indicated in the key. The I-N coexistence regions predicted by Chen [20] are shown as shaded regions, while DFT-CS results are shown as curves, and symbols are Monte Carlo simulations (diamonds [49]) and MD simulations (circles [43]), respectively. Here, MD data show as a simple transition density $\rho_{\mathrm{tr}}$ the inflection points of the *S* vs. *ρ* curves; (**b**) Same as (a), but choosing density and chain length *N* as variables. As in (a), shaded stripes are the two-phase coexistence regions predicted by Chen [20], full curves DFT-CS and symbols the MD data [43], for several choices of $\epsilon_{b}$: $\epsilon_{b}=8$ (blue); $\epsilon_{b}=16$ (green); $\epsilon_{b}=32$ (red); $\epsilon_{b}=100$ (purple, MD); $\epsilon_{b}=128$ (purple, DFT-CS). Reproduced from [43] with permission from the Royal Society of Chemistry.

The theory of Chen [20] only gives information on the variation of the transition densities $\rho_{i}$, $\rho_{n}$ as a function of the parameters $\ell_{p}$ and *L* and does not discuss the properties of the system in the nematically ordered phase further. Thus, we proceed next to a comparison of these predictions [20] to the MD and DFT results [42]; Figure 2. It is seen that for very stiff chains, both the theory of Chen [20] and DFT [42] are in reasonable agreement with the MD results, and nematic order still occurs for chains as short as *N* = 8. For relatively flexible chains ($\epsilon_{b}=8$), DFT and MD agree rather well, while the theory based on the second virial approximation [20] is far off, as expected, because the transition densities are rather large. At the same time, we see that DFT-CS predicts a spurious upturn of the transition densities with *N* for intermediate values of $\epsilon_{b}$ (specifically, $\epsilon_{b}$ = 16 and 32), which is not confirmed by MD [42].

**Figure 3:**
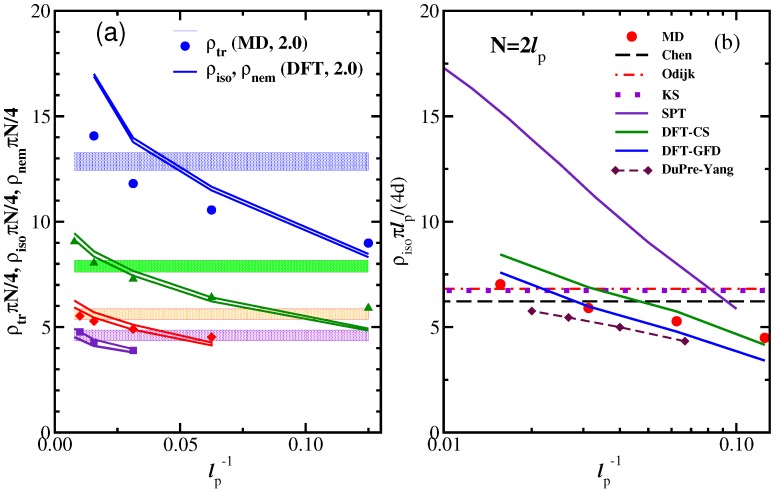
(**a**) Transition densities scaled as $\rho_{i}\pi N/4$, $\rho_{n}\pi N/4$ and $\rho_{\mathrm{tr}}\pi N/4$ plotted vs. $d/\ell_{p}$ (using units where d=σ=1), for four choices of $N/\ell_{p}$, distinguished by color: $N/\ell_{p}=2$ (blue); $N/\ell_{p}=\;1$ (green); $N/\ell_{p}=0.5$ (red); $N/\ell_{p}=0.25$ (purple). Symbols are the MD results; full curves denote DFT-CS predictions [43]; while the horizontal shaded stripes show the I-N coexistence regions from [20]. Note that $\rho L/d=\left(\rho \ell_{p}/d\right)L/\ell_{p}$ is plotted here rather than $\rho \ell_{p}/d$ discussed in the text, to avoid cluttering the figure, and the factor π/4 accounts for the normalization of the density with the cylinder volume $\ell_{p}d^{2}\pi /4$ as in the Onsager theory; (**b**) Transition density $\rho_{i}\pi \ell_{p}/\left(4d\right)$ plotted vs $\ell_{p}^{-1}$, comparing MD data to the theories of Chen [20], Odijk [16], Khokhlov and Semenov [12,13], scaled particle theory (SPT) [18,21], DFT-CS, DFT-generalized Flory dimer (GFD) and the DuPré–Yang theory [19], as indicated in the key. Reproduced from [43] with permission from the Royal Society of Chemistry.

As stated already in Equation (2) and can be deduced from Equation (23), noting that c≈ρ if the distinction between L/d and *N* is neglected (strictly speaking, $L=\left(N-1\right)\ell_{b}$, and we take $\ell_{b}\approx d\approx \sigma$ here), the scaled transition densities $\rho_{i}\ell_{p}/d$, $\rho_{n}\ell_{p}/d$ are functions of the ratio $\ell_{p}/L$ only. Hence, when one studies the variation of these transition densities with the parameter $d/\ell_{p}$ keeping the ratio $\ell_{p}/L$ fixed, one expects simply to find horizontal straight lines. However, Figure 3 shows that this is not the case: the scaled transition densities distinctly decrease with increasing $d/\ell_{p}$. This behavior is rather well described by DFT-CS for $N/\ell_{p}$ = 0.25, 0.5 and 1, while for $N/\ell_{p}$ = 2, the agreement is only qualitative. Replotting this latter case choosing a logarithmic rather than linear abscissa scale, we have also included predictions from the theories of DuPré and Yang [19] and Sato and Teramoto [18,21] for comparison. As has been discussed in Section 2.3, these theories renormalize the prefactor of the second virial term in Equation (23), to allow an extension of the description to higher densities and, thus, predict now a dependence of the transition densities on the parameter $d/\ell_{p}$ even when $L/\ell_{p}$ is constant. Figure 3b shows, however, that the variation predicted by the scaled particle theory [18,21] is far too strong, at least for the shown example. In this case, the theory of DuPré and Yang [19] is rather close to both DFT and MD results [42]. It is also shown that the differences between the theories of Khokhlov and Semenov [12,13,14], Odijk [15,16] and Chen [20] do not seem important, in comparison to the shortcoming that they do not yield any dependence on the parameter $d/\ell_{p}$ at all. There is also some disagreement between the two versions of DFT introduced here (Section 2.2); in view of the rather crude approximations that were necessary to introduce in the DFT framework, these discrepancies are not really surprising. We also mention that many of the corresponding experimental data (see [22] for a review) are believed to correspond to the regime where $0.005<d/\ell_{p}<0.03$. In this range, the deviations between the MD results [42] and Chen’s theory [20] are typically less that 15%. Since experimental data are hampered by polydispersity and by significant uncertainty regarding both parameters *d* and $\ell_{p}$ [22], very good quantitative agreement between theory and experiment cannot be expected. However, in those cases, when $d/\ell_{p}$ is not so small, the dependence of the transition densities on this parameter found in [42,43] should be relevant.

### 3.2. Nematic Order Described as an Effective Cylindrical Confinement

**Figure 4:**
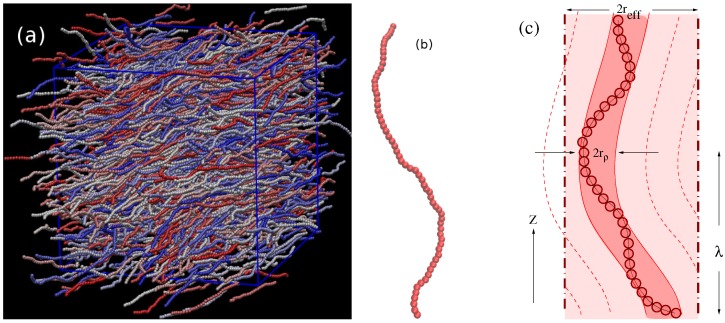
(**a**) Snapshot of a system of semiflexible polymers with length N=32, stiffness $\epsilon_{b}=100$, at concentration ρ=0.6 (with nematic order parameter S≈0.9). Chains are shown in different colors so that they can be better distinguished visually; (**b**) Typical conformation of a semiflexible polymer in the nematic phase ($N=64,\;\epsilon_{b}=16,\;\rho =0.4,\;S\approx 0.9$); (**c**) Schematic description of nematic order as effective cylindrical confinement: each chain has its own cylindrical bent tube of diameter $2r_{\rho }$ defined such that it contains only monomers from the considered chain. This tube roughly follows the contour of this macromolecule, which shows long wavelength undulations with a typical wavelength given by the deflection length. The typical amplitude of these deflections is of the order $r_{\mathrm{eff}}$ defining a cylinder (the straight axis of this cylinder is oriented along the director of the nematic phase). This cylinder contains not only a single bent tube, but rather is densely filled by a whole bundle of neighboring tubes whose deflections are strongly correlated. Reproduced from [42] with permission from the APS.

Here, we return to Figure 1c, where the nematic order parameter was plotted as a function of density. A remarkable feature is that the increase of S(ρ) from $S_{c}$ towards the ultimate saturation value S=1 predicted by DFT is much more rapid than according to MD, irrespective of how well the transition densities ($\rho_{i},\rho_{n}$) are predicted. A clue to this qualitative discrepancy is obtained from an examination of snapshot pictures of the chain configurations in the nematic phase (Figure 4). Even when the nematic order parameter is already large, the chains still exhibit considerable orientational disorder on large length scales. Superimposed on these large wavelength deflections, there are also some more or less random small-scale orientational fluctuations of the bond orientations relative to such a coarse-grained contour, which can be taken schematically as the axis of the bent tube of diameter $2r_{\rho }$ (see Equation (27)) shown doubly shaded in Figure 4c. Since the excluded volume interactions between monomers from different chains are strictly respected, we can define these bent tubes such that they contain monomers from the considered chain only. In the coordinate system along the tube contour, we then have (for N≫1):

(27) $$N=\rho Lr_{\rho }^{2}\pi ,\;\;r_{\rho }=1/\sqrt{\pi \rho \ell_{b}}.$$

Since for large *S*, the deflections of the tubes away from the *z*-axis (which we orient along the nematic director) can involve only small polar angles *θ*, we shall have $r_{\mathrm{eff}}\approx \lambda \theta$, where the deflection length *λ* is a characteristic length of the problem.

The deflection length *λ* is a concept well known for the problem of confinement of a single semiflexible chain in a cylinder with repulsive walls [51,52,53,54]. The deflection length concept means that the orientation correlation function 〈cosθ(s)〉 along a chain reaches a plateau as a function of *s*, because a chain confined in a cylinder is “deflected back” when it reaches the cylinder walls, and then, the angular mean-square displacement $\langle \theta^{2}\left(s\right)\rangle$ cannot increase any further. If one considers a regime where hairpin formation [95,96] can still be neglected, one can still apply Equation (7) for distances $s\ell_{b}<\lambda$, and we use $\langle \cos\theta \left(s\right)\rangle \approx 1-\frac{1}{2}\langle \theta^{2}\left(s\right)\rangle$ to conclude:

(28) $$\frac{1}{2}\langle \theta^{2}\left(s\right)\rangle =s\ell_{b}/\ell_{p}=\frac{1}{2}{\left(\frac{r_{\mathrm{eff}}}{\lambda }\right)}^{2},$$

and putting, $s\ell_{b}=\lambda$ one finds $\lambda ={\left(\ell_{p}r_{\mathrm{eff}}^{2}/2\right)}^{1/3}$. Since $S=\frac{3}{2}\langle \cos^{2}\theta \rangle -\frac{1}{2}\approx 1-3\langle \theta^{2}\rangle /2=1-\;3/2{\left(r_{\mathrm{eff}}/\lambda \right)}^{2}=1-\left(3/2^{1/3}\right){\left(r_{\mathrm{eff}}/\ell_{p}\right)}^{2/3}$, one concludes that $1-S\approx \left(3/2\right){\left(2r_{\mathrm{eff}}/\ell_{p}\right)}^{2/3}$. By a similar argument, one can estimate the *z*-component of the end-to-end vector of the (strongly stretched) chain in the cylindrical tube. One can consider the macromolecule as a sequence of essentially straight pieces of length *λ* with an average misorientation given by the factor $\langle \cos\theta \left(s\right)\rangle \approx 1-\frac{1}{2}\langle \theta^{2}\left(s\right)\rangle$. Roughly, the mean-squared end-to-end distance $\langle R_{e}^{2}\rangle$ will be reduced relative to $L^{2}$ by a corresponding factor, putting $\langle R_{e}^{2}\rangle \approx \langle |R_{ez}{|\rangle }^{2}\approx L^{2}\left(1-\langle \theta^{2}\rangle \right)$. Of course, such an order of magnitude estimates can be substantiated by more accurate theories [52,53,54] to yield:

(29) $$1-S=0.510{\left(2r_{\mathrm{eff}}/\ell_{p}\right)}^{2/3},\;\;1-{\langle R_{e}^{2}\rangle }^{1/2}/L=\left(1-S\right)/3.$$

Equation (29) was derived for a single semiflexible chain confined in a cylinder of radius $r_{\mathrm{eff}}$ with repulsive walls. The key idea of Odijk [15,16] and Egorov et al. [42,43] has been to postulate that the effect of the “nematic mean field” orienting the considered semiflexible macromolecule in the nematic phase can be described by a confinement in an effective cylinder. Thus, Equation (28) for $s\ell_{b}=\lambda$ implies a relation between the deflection length and the order parameter reduction, namely:

(30) $$\lambda =\frac{1}{2}\ell_{p}\langle \theta^{2}\rangle =\frac{1}{3}\left(1-S\right)\ell_{p}.$$

Odijk [16] also suggested that the concept of the deflection length implies chain-end effects on the local orientational order S(s) along the chain:

(31) $$S_{\infty }-S\left(s\right)\propto \exp\left[-sl_{b}/\lambda \right],$$

near s≈0 or s≈L, with $S_{\infty }$ being the local order far from the chain ends, for $L\gg \ell_{p}$.

**Figure 5:**
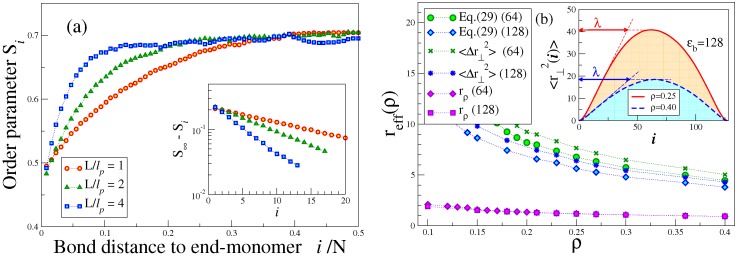
(**a**) Local order parameter $S_{i}$ that describes the orientation of the bond connecting monomers at $r_{i}$ and $r_{i+1}$ (the free ends being i=1 and i=N, and all equivalent bonds in the system are averaged over) plotted versus i/N for N=128, the total number of monomers being NN= 460,800, for three choices of $\epsilon_{b}$: $\epsilon_{b}/k_{B}T=128,\;\rho =0.1;\epsilon_{b}/k_{N}T=64,\;\rho =0.16$; $\epsilon_{b}/k_{B}T=32,\;\rho =0.25$. The densities were chosen such that the nematic order parameter *S* is close to 0.7 in each case. The three choices of the parameter $N/\epsilon_{b}$ (which roughly corresponds to $L/\ell_{p}$) are indicated in the key. The inset shows a semi-log plot of $S_{\infty }$-$S_{i}$ vs. *i* to test Equation (31). The resulting values of *λ* are 8.2, 3.65 and 2.36, respectively; (**b**) Effective cylinder radius $r_{\mathrm{eff}}$ plotted vs. density *ρ*, for *N* = 128, and two choices of $\epsilon_{b}$: $\epsilon_{b}=64$ and 128, as indicated in the key. Furthermore, the radius $r_{\rho }$ (Equation (27); cf. Figure 4c) is included for comparison. The inset shows a plot of the transverse mean-square displacements $\langle r_{⊥}^{2}\left(i\right)\rangle$, relative to the end-to-end vector of the chain, as a function of *i*, for $\epsilon_{b}=128$ at two densities, as indicated. Equation (28) implies that $\langle r_{⊥}^{2}\left(i\right)\rangle$ increases linearly with *i* and saturates at i≈λ with $\langle r_{⊥}^{2}\left(i\right)\rangle \approx \;{\left(r_{\mathrm{eff}}/\lambda \right)}^{2}$. The data points for $r_{\mathrm{eff}}$ are extracted from these maximum transverse displacements, and the estimates extracted from Equation (29) are included for comparison. Reproduced from [43] with permission from the Royal Society of Chemistry.

**Figure 6:**
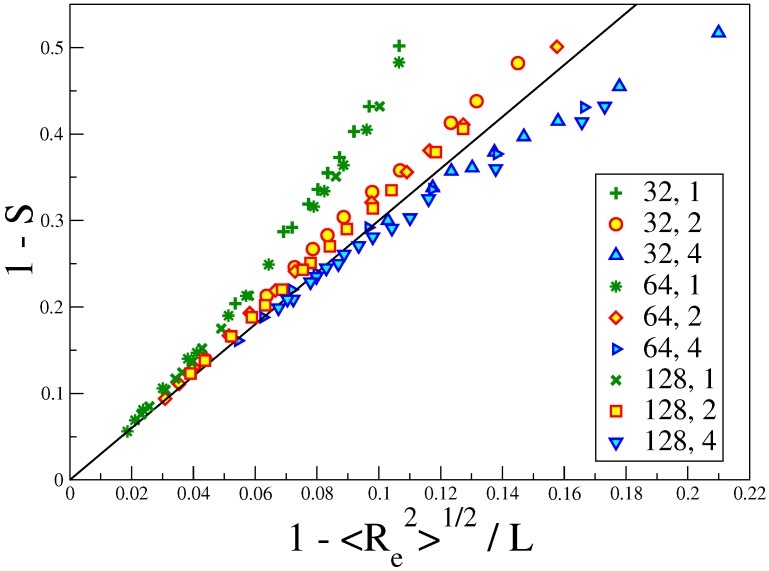
Plot of the order parameter reduction 1-S versus the relative reduction of the end-to-end distance $1-{\langle R_{e}^{2}\rangle }^{1/2}/L$. Three choices of the chain length *N* (N=32,64 and 128), and in each case, three choices of the parameter $N/\epsilon_{b}=1,\;2$ and 4 are included, as indicated in the key. The straight line indicates Equation (29). Reproduced from [43] with permission from the Royal Society of Chemistry.

Equations (29)–(31) were first tested by MD simulations by Egorov et al. [42,43], and we reproduce their key results in Figure 5 and Figure 6. Indeed, the description developed above is confirmed, at least qualitatively. Equation (31) is compatible with the data, and the resulting estimates of *λ* are roughly proportional to $\ell_{p}$ (but somewhat smaller than predicted by Equation (30)). Figure 5b shows that $r_{\mathrm{eff}}$ exceeds $r_{\rho }$ by far, for small densities, confirming the qualitative picture (Figure 4c); when *S* decreases (with decreasing density), the deflection length increases, and hence, also $r_{\mathrm{eff}}$ increases. The inset of Figure 5b shows that even for *N* = 128, the chains are too short to clearly display a pronounced horizontal plateau in the plot of $\langle r_{⊥}^{2}\left(i\right)\rangle$ vs. *i* in the middle part of the chains, and hence, the accuracy with which estimates for $r_{\mathrm{eff}}$ and *λ* can be extracted here is still limited.

A particularly interesting comparison is seen in Figure 6, which shows that for 1-S≪1, Equation (29) is quantitatively satisfied, for all *N* and $\epsilon_{b}$ values shown there, without any adjustable parameters. This implies that in the well-ordered nematic state, the picture of the ordering as effective cylindrical confinement is self-consistent. It is clear that for 1-S≥0.3, this picture gradually breaks down; we have used $\langle \theta^{2}\rangle \ll 1$ throughout, and this no longer holds then. Therefore, the description in terms of cylindrical confinement is no longer accurate in the nematic phase near the I-N transition (where 1-S≈0.5).

We conclude this picture by stressing that Figure 4c (which emphasizes $r_{\mathrm{eff}}\gg r_{\rho }$, as verified in Figure 5b) implies that there must occur collective deflection fluctuations of many neighboring chains, since the arrangement of the bent tubes with the diameter $2r_{\rho }$ is space filling and the tubes must not overlap each other. Such collective modes (with rather large wavelengths) are missing in the DFT descriptions of the ordered phase, of course: the picture is analogous to the molecular field theory of (classical) isotropic magnets, where the Langevin function also predicts a much faster saturation of order, rather than theories taking into account the long wavelength spin waves.

An intriguing question is to connect this description to the long wavelength description of fluctuations in the nematic in terms of the Frank elastic constants. Gemünden and Daoulas [97] succeeded in estimating the latter for a discretized worm-like chain model where bonds interact with a soft anisotropic potential.

It would also be very desirable if one would have experimental data on these issues to compare. X-ray scattering experiments from polymer nematic liquid crystals (such as poly-*γ*-benzyl glutamate in dioxane) have been performed and reveal very interesting information on the anisotropic character of density fluctuations [98], but cannot elucidate the nontrivial interplay with the fluctuations of orientational order.

## 4. Semiflexible Chains Confined by Repulsive Walls

The effect of repulsive walls on solutions of semiflexible polymers is somewhat subtle: the chains have reduced translational freedom near a wall, but their orientation parallel to a wall gets enhanced. The first effect dominates as long as the solution is relatively dilute, while the second effect will lead to local nematic order near the wall when enhancement of density enforces enough chains to be located close to the walls.

**Figure 7:**
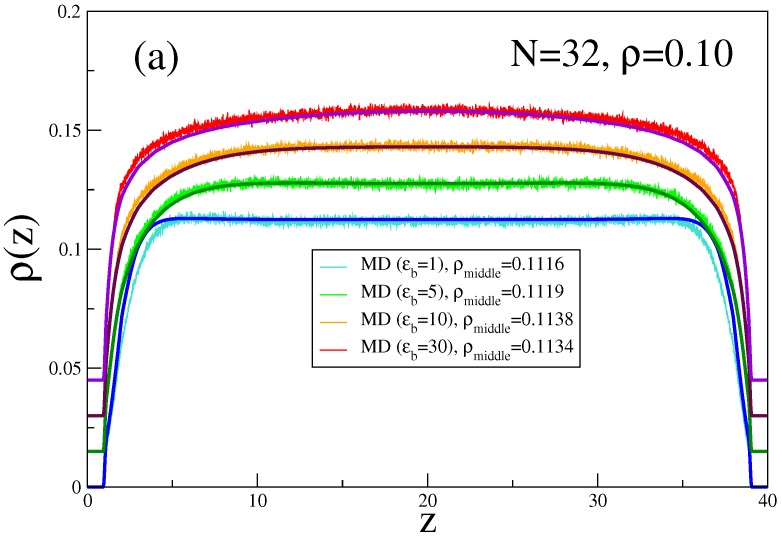

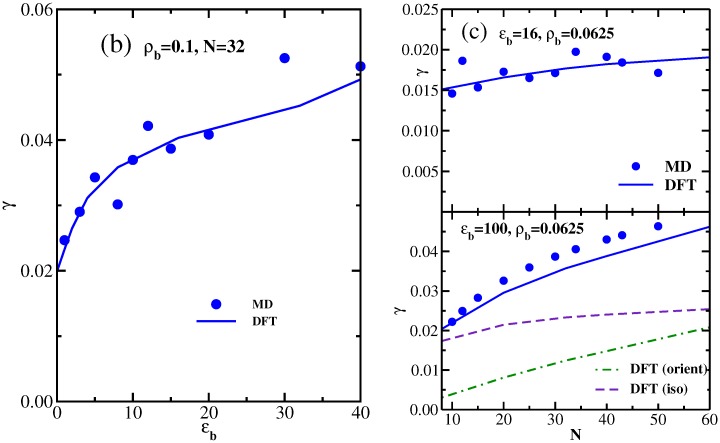
(**a**) Monomer density profiles ρ(z) across the film for $L_{z}=40$ and four values of the stiffness parameter $\epsilon_{b}=1,5,10$ and 30, choosing N=32 and ρ=0.1 in the MD simulation. The MD profiles are the noisy curves, while the corresponding DFT calculations were done choosing a chemical potential for which the bulk density $\rho_{b}$ coincides with the density $\rho_{\mathrm{middle}}$ in the middle of the film, at $z=L_{z}/2$. These densities $\rho_{\mathrm{middle}}$ are quoted in the key of the figure, and the smooth lines show the DFT profiles. Note that the curves are shifted vertically by 0.015 relative to each other for the sake of better visibility; (**b**) Surface tension *γ* (Equation (22)) plotted vs. $\epsilon_{b}/k_{B}T$ for the case $\rho_{b}=0.1$ and N=32, comparing MD results (dots) with DFT predictions (line); (**c**) Same as (b), but for the case $\rho_{b}=0.0625$, $\epsilon_{b}=16$ (upper part) and $\epsilon_{b}=100$ (lower part), plotted vs. chain length *N*. The contributions of the isotropic and orientational terms are shown as broken and dash-dotted curves, respectively. Reproduced from [43] with the permission of AIP Publishing.

These qualitative expectations are substantiated by the MD and DFT calculations of Egorov et al. [44]. Figure 7 gives an example for relatively short chains (*N* = 32) at ρ=0.10, where a choice of $L_{z}=40$ is enough to ensure that the system at distances near $z=L_{z}/2$ still exhibits bulk-like behavior. In this case, the MD simulations suffer from the obvious problem that MD is performed at a constant monomer density *ρ*, which is chosen beforehand, but in general differs from the bulk density $\rho_{b}$ (which is seen eventually in the middle of the slit, provided $L_{z}$ is large enough) by a correction of order $1/L_{z}$, due to the wall excess density. Of course, the choice of $L_{z}$ is somewhat arbitrary, and there clearly is interest in data that are not affected by a dependence on such a parameter (which drops out in the limit of a semi-infinite system, but the latter is not accessible by simulations). For a meaningful quantitative comparison between MD and DFT (Figure 7a), where the density $\rho_{b}$ corresponds to that of a bulk system in the grand canonical ensemble, Egorov et al. [44] chose the chemical potential of the DFT calculation, such that $\rho_{b}$ coincides with $\rho_{\mathrm{middle}}$ as observed in the MD simulation. One can see from Figure 7a that $\rho_{b}$ exceeds *ρ* by about 14% due to the negative surface excess density at the repulsive walls. However, when this readjustment is made, nearly perfect agreement between DFT and MD is noted. We also mention that MD cannot use the definition given by Equation (22) to estimate the surface tension, but rather one exploits the anisotropy of the pressure tensor (e.g., [99]) $p_{\alpha \beta }\left(z\right)$ [α,β=x,y,z]:

(32) $$\gamma_{\mathrm{wall}}=\frac{1}{2}\int_{0}^{L_{z}}dz\left[p_{zz}\left(z\right)-\frac{1}{2}\left(p_{xx}\left(z\right)+p_{yy}\left(z\right)\right)\right].$$

A nontrivial issue is the fact that the implementation of Equation (32) cannot follow the standard prescriptions [100] since $p_{\alpha \beta }\left(z\right)$ near the walls is strongly affected by the three-body forces deriving from the bending potential given by Equation (5) [101] even though these forces cancel out in the bulk. We also note (Figure 7a) that the bending potential, that distinguishes the semiflexible polymers from the flexible ones, leads to a much larger range of *z* near the walls over which the monomer density is depleted. Note also that for ρ=0.1, there is no indication yet of the oscillatory density profile near the wall (“layering”) that occurs at higher densities.

For simplicity, in Figure 7b,c, the distinction between $\rho_{b}$ and *ρ* is neglected, and since the chain models of MD and DFT differ slightly, it would be unrealistic to expect perfect agreement between the two methods (such as noted in Figure 7a) in general. However, both MD and DFT show the same qualitative trends, namely an increase of *γ* with either $\epsilon_{b}$ or *N*. Note that the observed agreement between MD and DFT can only be achieved provided that the DFT takes into account both the spatial density variations (contributing already to the isotropic part of *γ*) and the orientational effects (Figure 7c).

**Figure 8:**
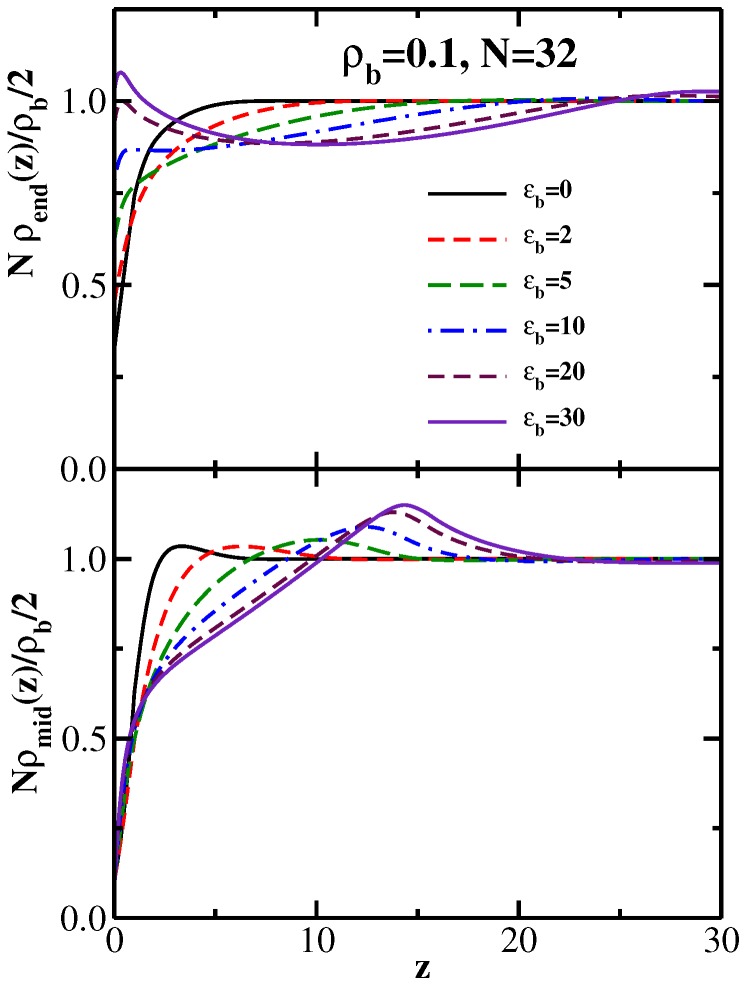
Normalized end-monomer (upper part) and mid-monomer (lower part) density profiles, $2N\rho_{\mathrm{end}}\left(z\right)/\rho_{b}$ and $2N\rho_{\mathrm{mid}}\left(z\right)/\rho_{b}$, plotted vs. *z*, for the case $\rho_{b}$ = 0.1, *N* = 32, and several choices of $\epsilon_{b}$, as indicated in the key. Reproduced from [43] with the permission of AIP Publishing.

The bonus of the DFT calculations is that very precise results can also be obtained for the density profiles of individual monomers, such as the densities of chain-ends or mid-monomers, respectively (Figure 8). These data are again in fair agreement with the corresponding MD results [44] (not shown here), but the latter suffer from strong statistical scatter.

For the low densities shown in Figure 8, there is not yet any trace of the familiar density oscillations (“layering”) near the repulsive walls that is found at distinctly higher densities, rather there is a clear depression of the density near the walls, and this depression extends more and more towards the film center when the chain stiffness increases. Thus, for low densities, indeed, there is no strong tendency in favor of local nematic order near the walls, but instead, there is a significant depletion of the density $\rho_{\mathrm{mid}}$ of the middle monomers of the chains near the walls over a range that increases with stiffness, followed by a maximum in their density profile further away from the walls.

**Figure 9:**
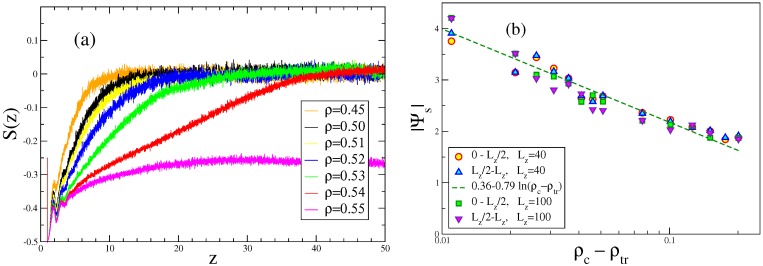
(**a**) Plot of the local order S(z) vs. *z* for the case N=8, $\epsilon_{b}=100$, $L_{z}=100$, where $\rho_{\mathrm{tr}}$ = 0.55, as obtained from MD; (**b**) Plot of the surface excess order parameter $|\Psi_{s}|$, with $\Psi_{s}=\int_{0}^{L_{z}/2}dzS\left(z\right)$, vs. $\rho_{\mathrm{tr}}-\rho$, for the case N=16, where $\rho_{\mathrm{tr}}$ = 0.30, using data for $L_{z}=40$ and $L_{z}=100$ (to check for finite size effects) and displaying data for $\Psi_{s}$ extracted from the range from z=0 to $L_{z}/2$, as well as from $z=L_{z}/2$ to $L_{z}$, to illustrate the large statistical scatter. The straight line illustrates the fit to a logarithmic variation, $|\Psi_{s}|=0.36-0.79\ln\left(\rho_{\mathrm{tr}}-\rho \right)$. Reproduced from [43] with the permission of AIP Publishing.

This behavior changes when we study densities close to the transition density $\rho_{\mathrm{tr}}$ of the bulk (Figure 9a). While S(z) (Equation (21)) in the isotropic phase far from the transition decays to zero rather fast, this decay gradually becomes slower as *ρ* approaches $\rho_{\mathrm{tr}}$, and very close to it, the decay occurs in two steps, indicating the formation of nematically-ordered layers attached to the walls. Right at $\rho =\rho_{\mathrm{tr}}$, the system is already well-ordered throughout the film (this “capillary nematization” [78] effect will be discussed below in more detail). This surface-induced ordering can be interpreted as the unbinding of an interface [102] between the nematic phase and the isotropic phase from the wall, and for short-range forces, between the wall and the interface (which are implied here because of Equation (9)), one predicts a logarithmic growth of the thickness of the nematically ordered layer. This thickness can be measured by the surface excess order parameter due to a wall,

(33) $$\Psi_{s}=\int_{0}^{L_{z}/2}dzS\left(z\right)=\int_{L_{z}/2}^{L_{z}}dzS\left(z\right),$$

as long as $S(z\approx L_{z}/2)=0$, so one still has the isotropic phase in the middle of the film. However, the isotropic-nematic interface is a mesoscopic, slowly fluctuating object, and hence, the statistical fluctuations of $\Psi_{s}$ when sampled from MD [44] or from Monte Carlo (MC) simulations [60,61,62] are huge. Both the MD data shown in Figure 9b and the earlier MC work [60,61] are compatible with the theoretically-expected variation [102]:

(34) $$\Psi_{s}\propto const-\ln\left(\rho_{\mathrm{tr}}-\rho \right),$$

but more precise data clearly would be desirable. The MC work [60,61,62] was based on the bond fluctuation model [103] for polymers on the simple cubic lattice, and this work suffers from the additional problem that the orientation of the director in the nematic layer can be only along the *x* or *y* axes of the lattice.

**Figure 10:**
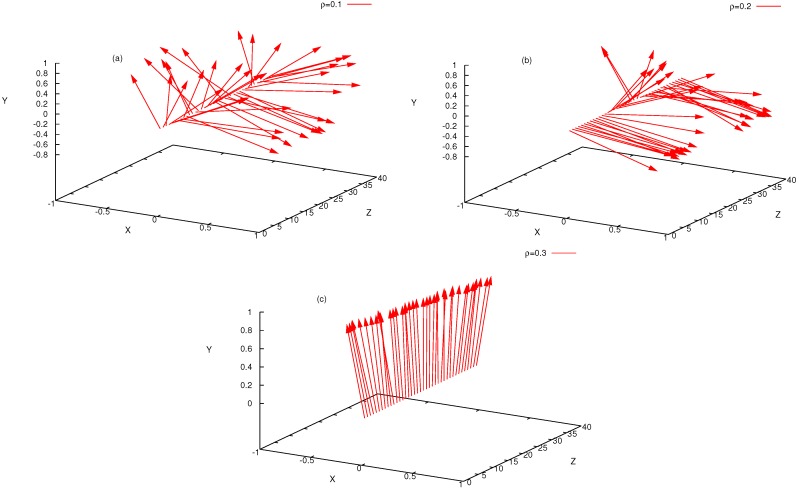
Plot of the layer-resolved director as a function of the *z*-coordinate across the film for the case N=32, N=1500 chains, $\epsilon_{b}=32$, $L_{z}=40,\;\Delta z=1.0$, and the densities ρ=0.1 (**a**); ρ=0.2 (**b**); and ρ=0.3 (**c**). The arrows show the orientations of the corresponding 40 unit vectors for each value of *z*. Note the different scales for *X*-, *Y*- and *Z*-directions. Reproduced from [45] with the permission of Wiley-VCH.

**Figure 11:**
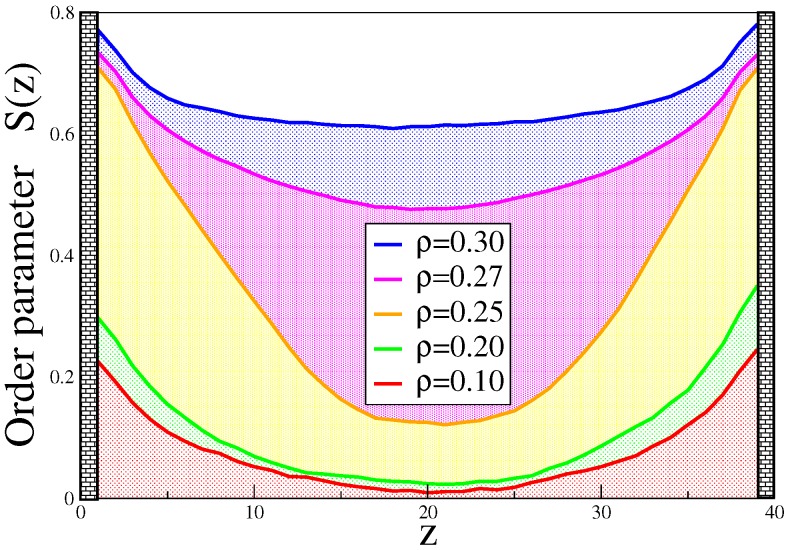
Thermally-averaged order parameter profile $\lambda_{+}\left(z\right)$ vs. distance *z* for the case $N=32,\;\epsilon_{b}=32,\;$ $L_{z}=40$, N=1500 and various densities, as indicated. Note that in the bulk, the I-N transition occurs at $\rho_{\mathrm{tr}}\approx 0.30$. Reproduced from [45] with the permission of Wiley-VCH.

The order parameter S(z) defined in Equation (21), where *θ* is the polar angle with respect to the *z*-axis, which was used in Figure 9, measures only the extent to which the bonds are oriented parallel to the wall. It does not measure the extent to which the bonds are aligned with a director. Using thus the local tensor $Q_{n,i}^{\alpha ,\beta }$ characterizing the orientation of a unit vector $u_{n,i}$ along the bond $r_{i+1,n}-r_{i,n}$ connecting monomers i,i+1 of the *n*-th chain,

(35) $$Q_{n,i}^{\alpha ,\beta }=\frac{1}{2}\left[3u_{n,i}^{\alpha }u_{n,i}^{\beta }-\delta^{\alpha \beta }\right],\;\;\left(\alpha ,\beta =x,y,z\right),$$

one finds a local director in a slice of width δz around *z* and averages $Q_{n,i}^{\alpha ,\beta }$ only over all of the bonds for which $r_{i,\mathrm{cm}}=\left(r_{i+1,n}+r_{i,n}\right)/2$ falls inside the slice. The largest eigenvalue $\lambda_{+}\left(z\right)$ then characterizes the proper local order (and the associated eigenvector is the local director). Figure 10 shows an example of the local directors in typical configurations of the system at three densities. One can see that at the lowest density ρ=0.1 for this case of rather stiff chains ($\ell_{p}=32$, N=32), whose end-to-end distance in the bulk is ${\langle R_{e}^{2}\rangle }^{1/2}=25.82$, and hence, of the same order is $L_{z}$, the arrows are more or less parallel to the walls, but in the xy-plane, their orientation is still rather random: although S(z) as defined from Equation (17) is close to -1/2 throughout the film, this clearly is not a nematically-ordered state. For ρ=0.2, on the other hand, there are several layers close to both walls where the directors are oriented parallel to each other, and only in the center of the film there still occurs a misalignment, as a remainder of a phase, which is still isotropic in d=2 dimensions. For ρ=0.3 (which corresponds to $\rho_{\mathrm{tr}}$ in the bulk), there is already a very high degree of order. This picture is substantiated when we record a thermally-averaged order parameter profile $\lambda_{+}\left(z\right)$; Figure 11. While for ρ≤0.2, there is only rather little order at the walls, for ρ=0.25, both walls are clearly coated by ordered layers. For ρ=0.27, the order parameter in the bulk is already about 0.5: capillary nematization has occurred. Near the bulk transition (ρ=0.3), the order in the film is clearly larger than it would be in the corresponding bulk system (Figure 1c).

We add a caveat: the observation of quasi-two-dimensional long-range nematic order implied by this discussion of Figure 11 could be just a finite size effect. As is well-known, the existence of nematic order in d=2 dimensions is a controversial issue (analogous to the Kosterlitz–Thouless transition [104] of two-dimensional XY-ferromagnets, there could be a state with a power law decay of orientational correlation functions, rather than true long-range order; see, e.g., [105,106]). In contrast, the lattice Monte Carlo simulations of Ivanov et al. [60,61,62] did find a well-defined order-disorder transition of the quasi-two-dimensional wall-attached layers, but due to the only two discrete director orientations, this is an Ising model-like transition and not a faithful representation of the nematic order of real semiflexible polymers.

**Figure 12:**
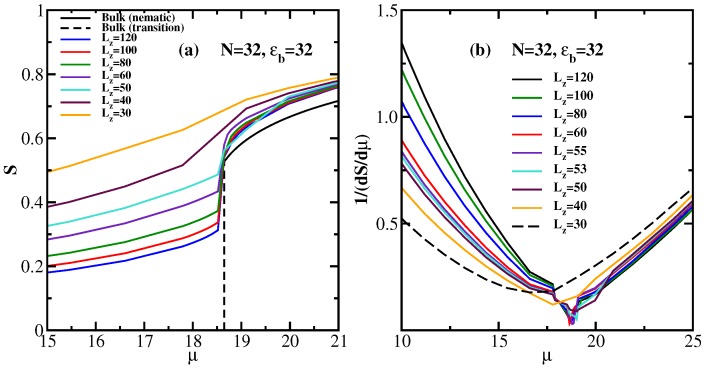
(**a**) DFT results for the nematic order parameter *S* as a function of the dimensionless chemical potential *μ* for $\epsilon_{b}=32$, N=32 and several choices of $L_{z}$, as indicated. The bulk behavior is included (the vertical broken line indicates the transition in the bulk); (**b**) Inverse response function ${\left[dS\left(\mu \right)/d\mu \right]}^{-1}$ plotted vs. *μ* for the same case as (a). Reproduced from [45] with the permission of Wiley-VCH.

Finally, Figure 12 shows corresponding DFT results. One can clearly see that for thin films, *S* is never strictly zero, due to the wall-induced order. For small values of $L_{z}$, such as $L_{z}=30$ or $L_{z}=40$, the variation of *S* with *μ* is clearly nonsingular, increasing *μ* (or increasing the density) simply causes a gradual onset of order, without a sharp transition. Egorov et al. [45] concluded that near $L_{z}=L_{z}^{*}\approx 55$, a capillary critical point occurs, and for $L_{z}>L_{z}^{*}$, there is a first-order transition (from a state with a smaller nematic order parameter to a state with a larger order parameter). Of course, due to its mean-field nature, DFT cannot answer the questions about the character of two-dimensional long-range nematic order.

It is encouraging that first experimental results on capillary nematization of colloidal rods under confinement have very recently become available [107], and we hope that the present article will stimulate corresponding work on confined semiflexible polymers.

## 5. Summary

In this review, recent theoretical and simulation work on the isotropic-nematic transition and the nematic order of semiflexible polymers was reviewed, considering both MD simulations and DFT calculations, but earlier theoretical work by Khokhlov and Semenov, Odijk, Chen and others [12,13,14,15,16,17,18,19,20,21], was also briefly included in the discussion. Only semiflexible polymers in lyotropic solutions were discussed, where the effective interactions between the monomeric units are short-ranged and repulsive in character, representing effectively the excluded volume of these units. Solvent molecules are not explicitly considered, and as in previous theoretical work [12,13,14,15,16,17,18,19,20,21], no attention was payed to the detailed atomistic structure and the corresponding potentials (e.g., torsional potentials etc.); and also, electrostatic forces were disregarded throughout. Thus, the stiffness of the semiflexible polymers was dealt with on a coarse-grained level, namely via the local persistence length $\ell_{p}$ (or the corresponding energy parameter $\epsilon_{b}$ of the bending potential between subsequent effective bonds along the chain, cf. Equations (5) and (6)). The repulsive monomer-monomer interaction is characterized by another characteristic length, the effective diameter *d* of the resulting worm-like chain, or (equivalently) the diameter σ=d of the effective monomeric units, which were modeled via the potential $U^{\mathrm{WCA}}\left(r\right)$ (see Equation (4)) in the MD framework, or by hard spheres in the DFT framework. Chain connectivity was modeled by anharmonic springs (described by $U^{\mathrm{FENE}}\left(r\right)+U^{\mathrm{WCA}}\left(r\right)$) in the MD work and by requiring the hard spheres to be tangent in the DFT work, while much of the earlier theories [12,13,14,15,16,17,18,19,20,21] were based on the continuum version (Equation (24)) of the Kratky–Porod model, where chain interactions then are described like in Onsager’s theory for long and thin hard rods [24] via the second virial coefficient [12,13,14,15,16,20] or modifications thereof [17,18,19,21]. We have not reviewed here the early simulation work (e.g., [46,47,48,49,50]), since most of this work dealt with comparatively short chains and relatively small systems, less suitable to characterize the I-N transition and the character of the nematic phase, but we have included results from early work (e.g., [49]) where appropriate. Both the behavior in the bulk solution and the effect of confinement by repulsive planar walls was considered.

It was shown that the scaled density where the I-N transition takes place shows a distinct dependence on both dimensionless parameters $L/\ell_{p}$ and $d/\ell_{p}$, and not only on $L/\ell_{p}$ alone, as the theories of Khokhlov and Semenov, Odijk and Chen [12,13,14,15,16,20] imply. DFT can account for this additional dependence on $d/\ell_{p}$ rather well, for typical cases that were studied, while the scaled particle theory [18,21] strongly overestimates this dependence on $d/\ell_{p}$. As expected, for $d/\ell_{p}$ less than 0.01, this dependence on $d/\ell_{p}$ becomes weak, and the approximations based on the second virial truncation [12,13,14,15,16,20] then become reasonably accurate. On the other hand, DFT predicts a spurious upturn of the transition density studied as a function of *L* for intermediate values of $\ell_{p}$, which is not confirmed by the simulations. Particularly drastic approximations are needed when one develops DFT to study the behavior of semiflexible chains near hard walls: it is necessary to neglect the coupling between the spatially inhomogeneous density profile and the orientational interaction term (Equation (20)), which needs to be inferred from the bulk (and there it needs to be taken from dedicated two-chain MC simulations [41]). Obviously, the DFT formulation for semiflexible polymers cannot yet be cast in the form of a completely self-contained analytical theory, it is still based on somewhat heuristic approximations. Nevertheless, the comparison with the MD results shows that particularly at high densities, it performs significantly better than the other theories. However, in the nematically-ordered phase, it predicts too large values of the nematic order parameter. This problem can be attributed to the neglect of collective deflection modes of the chains relative to the director. These deflections can be understood in terms of the analogy between semiflexible chains confined in cylindrical tubes and in the nematic phase.

A key point of our description is (Figure 5b) that the deflection length *λ* is not related to the perpendicular monomer displacements of order $r_{\rho }$ (nearest monomer distance in the plane perpendicular to the director), but a much larger length $r_{\mathrm{eff}}$ due to collective chain bending, contrary to naive expectations [16,98].

When the effect of repulsive walls is considered, one finds that for small monomer densities, the conformations of chains located near the wall are strongly deformed, leading to an enhancement of chain-end densities near the walls, while the middle monomers are depleted near the wall and are more likely to be found away from it. At larger densities, one finds that a wall-induced nematic order sets in. Of course, only a rough estimate could be given of the capillary nematization critical point, and a more complete variation of all of the parameters ($\ell_{p},L$) for this problem is still lacking. No attempt to clarify the nature of the quasi-two-dimensional phase in thin film geometry could be made.

Egorov et al. [42,43] also have suggested that the results reviewed here may also help to better understand the available experimental data on the I-N transition of semiflexible polymers, if one takes the predicted dependence on the parameter $d/\ell_{p}$ into account. Unfortunately, these parameters *d* and $\ell_{p}$ are known only rather imprecisely, and hence, it is difficult to draw firm conclusions on the experiments. Thus, it remains a challenge to improve the database on which a really conclusive comparison between experiment and theory can be based. Future work could also consider the effect of confinement by planar walls with attractive interactions, spherical confining surfaces, etc. Finally, we note that the focus of the present review is on the isotropic-nematic phase behavior of semiflexible polymers. In this regard, it is important to mention that the effect of stiffness on the nematic-smectic phase transition of semiflexible polymers is another interesting problem, which has been actively studied both experimentally and theoretically [108,109,110,111]. Thus, calculating more complete phase diagrams by including also the smectic phase would be of interest and could be considered in future research.
